# Drug-Resistant (DR) Tubercular Pleural Effusion: A Rare Case

**DOI:** 10.7759/cureus.31185

**Published:** 2022-11-07

**Authors:** Juhi Kadukar, Gaurang M Aurangabadkar, Pankaj Wagh, Ulhas Jadhav, Babaji Ghewade, Mrinmayee V Mayekar, Puja Upadhyay

**Affiliations:** 1 Respiratory Medicine, Jawaharlal Nehru Medical College, Datta Meghe Institute of Medical Sciences, Wardha, IND; 2 Respiratory Medicine, Datta Meghe Medical College, Datta Meghe Institute of Medical Sciences, Wardha, IND

**Keywords:** multi-drug resistant tuberculosis, genexpert mycobacterium tuberculosis/rifampin assay, line probe assay, bedaquiline, drug-resistant tuberculosis

## Abstract

Tuberculosis (TB) is one of the most common infectious diseases in developing countries throughout the world. According to the WHO, there has been a rise in the number of cases of drug-resistant (DR) TB in recent times. Tubercular pleural effusion is challenging to diagnose given the low bacillary load and frequently negative stains for acid-fast bacilli (AFB) on Ziehl-Neelsen (ZN) staining. We present a case of successful diagnosis and management of primary extra-pulmonary multidrug-resistant (MDR) tubercular pleural effusion after being misdiagnosed from outside as drug-sensitive extra-pulmonary TB. Initial tests revealed exudative effusion with raised adenosine deaminase (ADA) levels, therefore the patient was started on conventional anti-tubercular therapy with isoniazid (H), rifampicin (R), pyrazinamide (Z), and ethambutol (E), but the patient did not improve in spite of regular treatment for two months, which warranted further investigations. Therefore Xpert® MTB/R assay (Cepheid Inc., Sunnyvale, USA), line probe assay (LPA), and drug sensitivity testing (DST) of the pleural fluid were sent, which were suggestive of R- and H-resistant tubercular effusion. The patient was started on an oral bedaquiline-containing regimen as per the WHO guidelines and the patient showed considerable improvement on follow up.

## Introduction

Tuberculosis (TB) is one of the major infectious diseases in developing countries throughout the world. Tubercular pleural effusion is caused by Mycobacterium tuberculosis (MTB). It is characterized by chronic intense accumulation of inflammatory cells and fluid in the pleural space [1]. In non-endemic areas, the incidence of pleural involvement in TB ranges from 3% to 5%, and in endemic areas is up to 30% [2]. The diagnosis is complex and thoracocentesis needs to be done multiple times and usually reveals an exudative, lymphocytic pleural effusion with high pleural fluid adenosine deaminase (ADA) levels in about 90% of the patients. However, the direct visualization of acid-fast bacilli (AFB) is less than 10% [3], adding to further diagnostic delays. It is, therefore, important that in all suspected cases of tubercular pleural effusion, there must be a comprehensive diagnostic strategy that should include Xpert® MTB/RIF assay (Cepheid Inc., Sunnyvale, USA), a nucleic acid amplification test and line probe assays (LPAs) to look for patterns of drug resistance, as highlighted in this case report of a young male patient who was initially diagnosed with drug-sensitive TB pleural effusion and showed poor response to conventional anti-tubercular therapy, and on further pleural fluid investigations revealed drug-resistance to both isoniazid (H) and rifampicin (R).

## Case presentation

**Figure 1:**
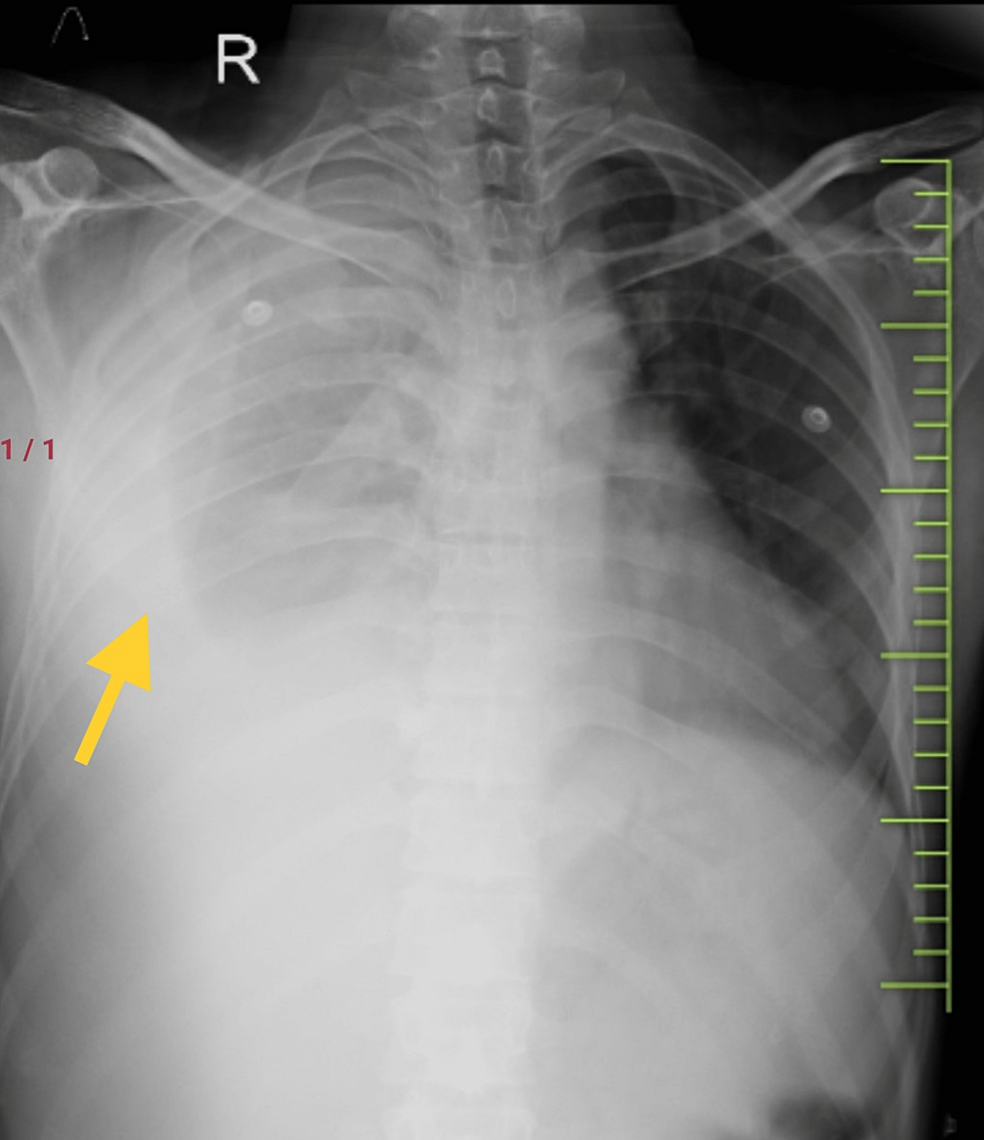
Chest x-ray posteroanterior (PA) view on admission showing right-sided massive pleural effusion (yellow arrow) with a contralateral displacement of the trachea and mediastinum

A 39-year-old male with no significant comorbid conditions presented to the emergency department, with complaints of right-sided pleuritic chest pain and dry cough for four months with evening rise of temperature which was low-grade in nature for three months. The patient also had a history of weight loss (more than 6 kg within a duration of two months) with generalized weakness. The patient also had gradually progressive dyspnea on exertion for two months. The patient initially went to a local government hospital, where his chest x-ray was done which was suggestive of massive right-sided pleural effusion with shifting of the mediastinum and trachea to the contralateral side (Figure 1 ).

Intercostal chest drain (ICD) insertion was carried out on the right hemithorax in view of the massive pleural effusion and all routine pleural fluid investigations were sent, summarized in Table 1.

**Table 1:** Summary of routine blood and pleural fluid investigations of the patient

INVESTIGATIONS	PATIENT VALUES	REFERENCE RANGE
Total leucocyte count (TLC)	12,000 cells per microlitre	4,500-11,000 cells per microlitre
Hemoglobin	10 grams percent	14-17 grams percent
Pleural fluid protein	5.3 grams per decilitre	1-2 grams per decilitre
Pleural fluid lactate dehydrogenase (LDH)	2,000 units per liter	Greater than 1,000 units per liter
Pleural fluid glucose	70 milligrams per decilitre	Less than 60 milligrams per decilitre
Pleural fluid adenosine deaminase (ADA)	50 international units (IU) per liter	Less than 40 IU per liter

**Figure 2:**
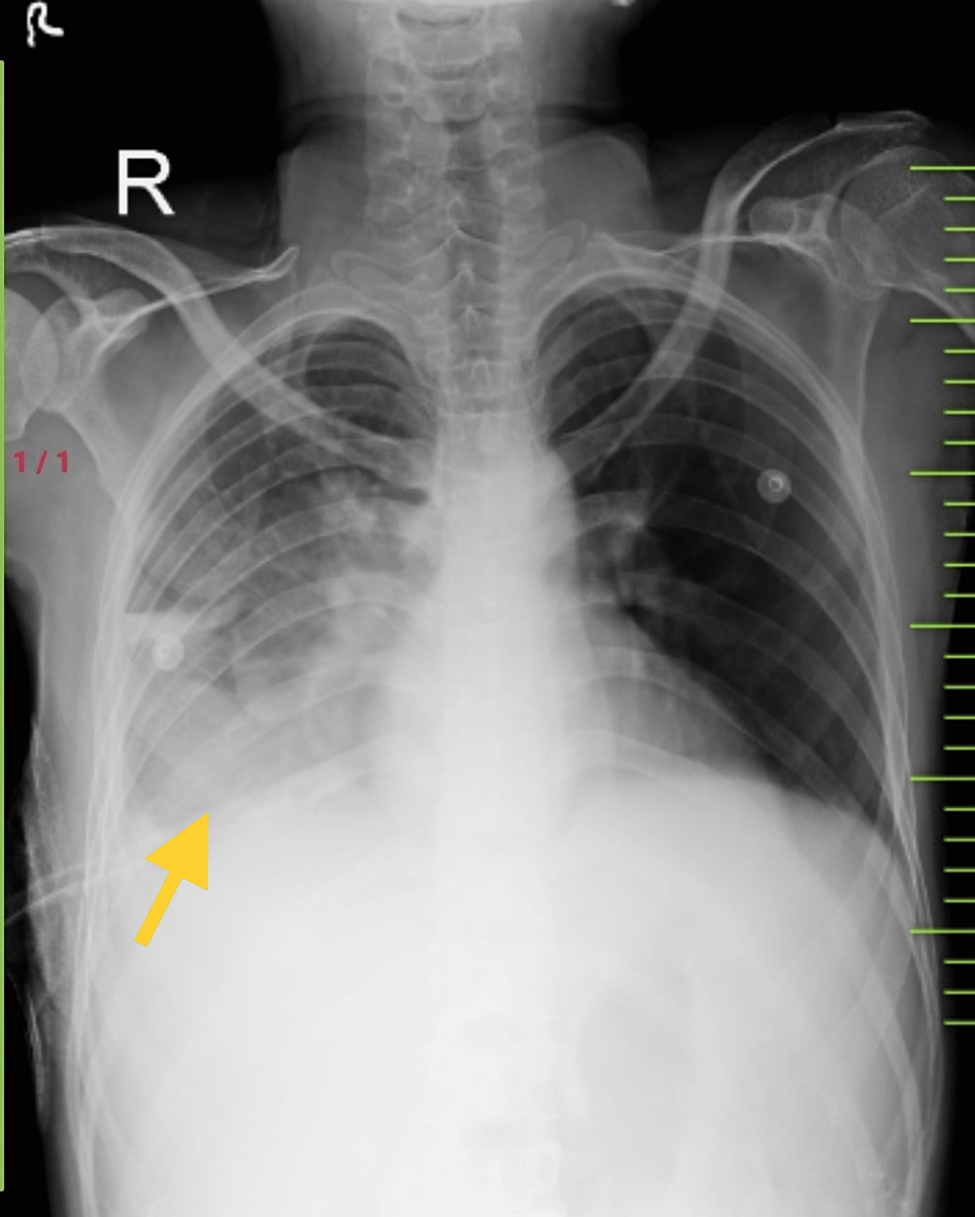
Chest x-ray posteroanterior (PA) view done after insertion of intercostal chest drain (ICD) on the right side showing significant resolution of the pleural effusion (yellow arrow)

A chest radiograph was again done post-ICD insertion on the right side which showed resolution of the pleural effusion with the return of the trachea and mediastinum to the midline position (Figure 2).

**Figure 3:**
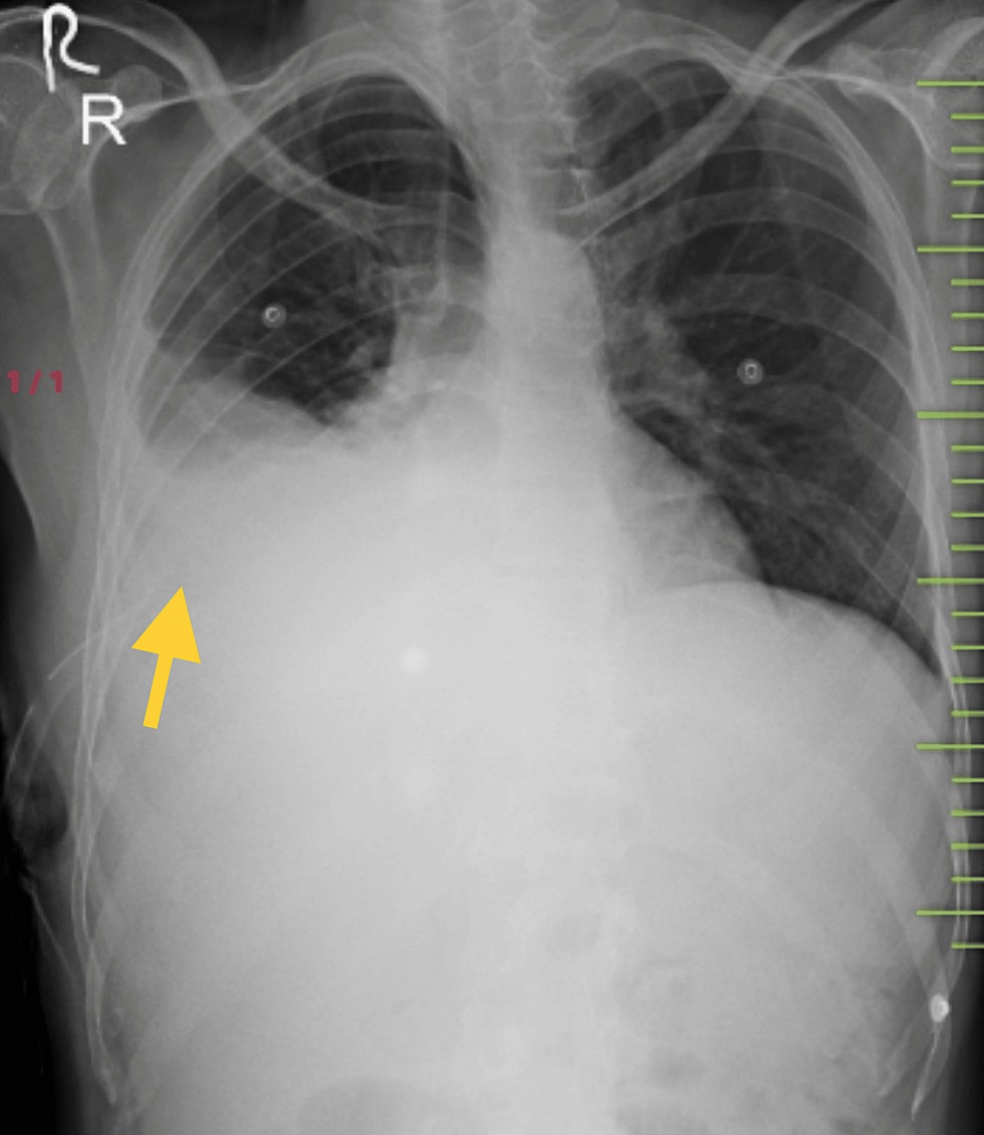
Chest x-ray posteroanterior (PA) view showing recurrence of the right-sided pleural effusion (yellow arrow) after two months of standard anti-tubercular regimen

The pleural fluid was found to be exudative in nature as per Light's criteria, fluid cytology was negative for malignant cells, and ZN staining of the pleural fluid was negative for AFB [1]. The patient was started on standard antitubercular therapy with H, R, pyrazinamide (Z), and ethambutol (E) on the basis of pleural fluid reports and was asked to follow up after two months. However, the patient did not report any clinical improvements after 2 months of standard anti-tubercular therapy [4]. A repeat chest x-ray showed a refilling of the right-sided pleural effusion (Figure 3).

Due to the absence of any significant clinical and radiological improvement in spite of the standard anti-tubercular regimen, repeat thoracocentesis was done and pleural fluid was sent for Xpert® MTB/RIF assay and LPA, which revealed the presence of MTB with resistance to both H and R. The patient was started on an oral bedaquiline-containing drug-resistant TB regimen and showed clinical and radiological improvements on follow up after one month [4].

## Discussion

India is the leading country in the world with respect to the number of active TB patients with an increasing number of drug-resistant TB patients being detected in the last decade. In India, the prevalence of multidrug-resistant TB (MDR TB) is 2-3% among new cases and 12-17% among reinfection cases [4]. The National Tuberculosis Elimination Programme presents five categories of DR TB: H-resistant TB, R-resistant TB, and MDR TB (R and H resistant), plus pre-extensively drug-resistant TB (pre-XDR TB), and XDR TB [4]. Causes of DR TB include poor adherence to treatment, delay in diagnosis, inadequate supply of drugs, low-quality medicines, unsupervised therapy, improper follow ups, and lack of education [5]. There is a lack of data on the primary extrapulmonary DR TB patients. Therefore, we report a case of an extrapulmonary drug-resistant tubercular pleural effusion. A review of the literature reveals that drug-resistant TB was present in 11 out of 103 patients diagnosed with tubercular pleural effusion in one study, while a separate study done in South Africa concluded a higher prevalence of drug-resistant extrapulmonary TB in the pediatric population who were found to be HIV seropositive [6,7]. It is, therefore, essential to also do an HIV screening of all patients with extrapulmonary TB and vice-versa [8]. Early diagnosis of drug resistance in extrapulmonary TB is of the utmost importance, as highlighted by our case that clinical and radiological improvement is not possible without starting an appropriate anti-tubercular drug regimen, tailored to each individual patient, as per their drug resistance patterns. A comprehensive diagnostic strategy with an appropriate drug regimen can reduce the development of serious pulmonary sequelae and complications in these patients of tubercular pleural effusion.

## Conclusions

All patients with MTB pleural effusion demonstrating poor response to therapy need pleural fluid reassessment for drug resistance by sending samples for Xpert® MTB/RIF assay and LPA. In TB-endemic countries like India, the drug resistance assay should be done in the primary sample analysis. This is especially important given the high TB burden in a country such as India. As highlighted by our case report, it is recommended that Xpert® MTB/RIF assay and LPA should be done as a primary investigation in all suspected cases of tubercular pleural effusion, in order to avoid unnecessary diagnostic delays and early initiation of effective anti-TB treatment. Also, drug sensitivity testing (DST) should be done on the pleural fluid to look for further patterns of drug resistance to both first and second line anti-TB drugs. It is, therefore, important to have a high index of suspicion for drug-resistant tubercular pleural effusion, especially in patients who are non-responders to standard anti-TB regimens.
